# Comparison of the Prescribing Pattern of Bisphosphonate and Raloxifene in Korean Women with Osteoporosis: From a National Health Insurance Claims Database

**DOI:** 10.1371/journal.pone.0127970

**Published:** 2015-06-01

**Authors:** Jungmee Kim, Ju-Young Shin, Joongyub Lee, Hong-Ji Song, Nam-Kyong Choi, Byung-Joo Park

**Affiliations:** 1 Department of Preventive Medicine, Seoul National University, College of Medicine, Daehangno, Jongno-gu, Seoul, Korea; 2 Korea Institute of Drug Safety and Risk Management, Boryung building, Changgyeonggung-ro, Jongno-gu, Seoul, Korea; 3 Medical Research Collaborating Center, Seoul National University Hospital and Seoul National University College of Medicine, Seoul, Korea; 4 Department of Family Medicine, College of Medicine, Hallym University, Chuncheon, Korea; University of Florida, UNITED STATES

## Abstract

This study aimed to evaluate the differences of prescribing pattern between bisphosphonate and raloxifene users among Korean women with osteoporosis, focusing on the underlying conditions, concurrent medications, nature of healthcare utilization, and regional disparity. We used the Health Insurance Review and Assessment Service National Patients Sample database of the year 2010. Study subjects were defined as female osteoporosis patients aged over 50 years with both the diagnosis of osteoporosis and prescriptions of bisphosphonate or raloxifene. The frequency and the proportion of bisphosphonate and raloxifene were compared using chi-square test and the trend of the proportion using the Cochran–Armitage test. Medications were quantified as defined daily doses per 1,000 patients per day. The prescription pattern was visualized by using the Quantum Geographic Information Systems program. Of the 1,367,367 people who utilized medical services in 2010, the final number of study subjects was 26,881—26,032 (96.8%) bisphosphonate and 849 (3.2%) raloxifene recipients. Raloxifene users were younger than bisphosphonate users and were more frequently patients with a lipid disorder (16.0% vs. 22.1%, p-value < 0.0001), rheumatic disease (4.0% vs. 6.1%, p-value = 0.0024), hot flash (1.8% vs. 6.1%, p <0.0001), and coronary artery disease (1.2% vs. 2.8%, p< 0.0001). The proportion of raloxifene users was higher in tertiary care institutions (21.6% vs. 44.7%, p-value < 0.0001). A regional distribution showed that raloxifene use was higher in the Seoul metropolitan area. These differences in demographic and clinical profiles of each recipient may influence prescription decisions.

## Introduction

Osteoporosis is a serious public health problem, particularly in postmenopausal women. In Korea, the prevalence of osteoporosis has been reported to be 33.3% among females aged over 50 years in the national health insurance claims data for 2005 and 2008 [1], and 35.5% in the data for 2008 and 2009, as obtained from the Korean National Health and Nutrition Examination Survey (KNHANES) [2]. Thus, one in three women in Korea is likely to have weakened bones with an increased risk of fractures. According to the Statistics Korea, a government organization for statistics, the number of individuals older than 65 years of age is predicted to reach 14.3% by 2018, which is the rapidest aging of any national population in the world. Therefore, the prevalence of osteoporosis in elderly women in Korea is expected to rise, while currently, Korea is categorized as a country with a medium risk of hip fracture as compared to 62 other countries [3].

Bisphosphonate and raloxifene are the osteoporosis medications most commonly prescribed by clinicians. While bisphosphonate is in the lead in its use, its long-term risks such as atypical fracture or osteonecrosis of the jaw are a concern [4]. The fracture risks, with respect to the bone turnover markers or bone mineral density (BMD), of these two drugs have been compared in several clinical trials [5–7] and observational studies using claims databases [8–10]. However, while bisphosphonate decreased the fracture incidence more in most of the studies, raloxifene had beneficial effects on lipid metabolism and breast cancer [5,11]. While bisphosphonates and raloxifene share a common mechanism of antiresorptive therapy, bisphosphonates suppress the osteoclast metabolism and raloxifene decreases bone degradation, resulting in the development of a combination of the two [12]. The most appropriate selection of osteoporosis treatment for individual patients remains controversial; therefore, the different efficacy and safety features of the two medications should be considered to optimize the treatment of osteoporosis patients.

Previous drug utilization studies have shown that raloxifene users are younger and in a better overall health condition than bisphosphonate users [13,14]. However, these studies were conducted by only one research group by using the United States claims database, and there is a lack of published data, focusing on the Asian population, on the prescription pattern of the two drugs. Therefore, this study aims to describe the prescription pattern of bisphosphonates and raloxifene in Korean women with osteoporosis in order to identify the demographic and clinical characteristics of the two drug users in actual clinical practice.

## Materials and Methods

### Database

In 1989, the Korean government achieved universal healthcare insurance coverage, which currently covers 97% of the total population of 50 million. The suitability of reimbursement of all the medical claims including prescriptions submitted by individual health care providers is reviewed by the government agency called the Health Insurance Review and Assessment (HIRA). In such a process, a database of national healthcare utilization is cumulated with data such as age, gender, healthcare provider characteristics, diagnoses using the International Classification of Diseases and Related Health Problems 10th revision (ICD-10) codes, procedures or operations, drug claims, type of insurance, and medical care costs. This HIRA database has been used in several epidemiologic studies in the field of osteoporosis [1,15,16]. HIRA provides the National Patients Sample (HIRA-NPS) database, which contains 3% of the national population representing the total Korean population of 50 million through its internet site (http://www.hira.or.kr/eng/index.html#&panel1-2). The nationwide health insurance database is first stratified into two strata regarding gender and 16 strata by age with 5 year interval, and then 3% is systematically random sampled from each stratum. The representativeness and the validity of this sample database have been confirmed by comparing estimates from the sample data and the whole population [17]. HIRA-NPS of the year 2010 with a total sample size of 1,367,367 was used in the analysis since this was the year with no change in the reimbursement criteria regarding osteoporosis treatment in Korea. The data underlying the present study is freely available upon request from the authors, and the raw data can be purchased from the Health Insurance Review and Assessment Service of Korea (http://www.hira.or.kr/eng/index.html#&panel1-2).

### Ethics statement

In the HIRA database, the claim records of each patient are anonymously linked using de-identified codes, making it impossible to identify them in the real world. Therefore, the present study has been exempted from review by the Institutional Review Board of Seoul National University Hospital and Seoul National University College of Medicine (IRB No: 1309-007-516).

### Study subjects and drugs

Inclusion criteria were women aged over 50 years who had been prescribed with bisphosphonates or raloxifene at least once with the diagnosis of osteoporosis by clinicians in 2010. The ICD-10 codes used for the selection of osteoporosis patients were M80 (osteoporosis with pathologic fracture), M81 (osteoporosis without pathologic fracture), or M82 (osteoporosis with multiple myeloma, endocrine disorders, etc.). Since the HIRA database only contains the claims of the reimbursed prescriptions, this claims database includes the prescriptions of patients that already met the criteria for reimbursement, which was T-score ≤ -3 in BMD for bisphosphonate or raloxifene. Therefore, all the study subjects are considered to have received bisphosphonates or raloxifene for treatment of osteoporosis, more than prevention, and have a high risk for fracture. Five kinds of bisphosphonates including alendronate, risedronate, ibandronate, pamidronate, and zoledronate along with one selective estrogen receptor modulator, raloxifene, were available for the indication of osteoporosis. Alendronate is given daily or weekly, while risedronate is given daily, weekly, twice in a month, or monthly. Ibandronate is given monthly, pamidronate daily or once in three months, and zoledronate yearly. In case of ibandronate, pamidronate, and zoledronate, some medications were administered intravenously. In Korea, it is only allowed to prescribe either one of the bisphosphonates or raloxifene in order to get covered by reimbursement in the national health insurance system, and therefore, there was no patient prescribed with both medications at the same time.

### Definition and measures of covariates

Comorbidities were defined as coexisting when any of their diagnoses were coded at least once during the period from January 1, 2010, to December 31, 2010. The prevalent comorbidities of the elderly such as chronic gastritis/gastroesophageal reflux disease (GERD), chronic low back pain, and osteoarthritis were described [18]. The underlying conditions reported to be susceptible to osteoporosis were referred to as endocrine disorders, chronic obstructive lung disease, rheumatologic and autoimmune diseases, depression, gastrointestinal disorders, end-stage renal disease, and hypogonadal states [19]. The comorbidities vulnerable to falling such as stroke, dementia, Parkinson’s disease, and epilepsy were also included [19]. Medications, such as proton pump inhibitors, selective serotonin reuptake inhibitors, anticonvulsants, glucocorticoids, thyroid hormones, and thiazolidinediones, known as risk factors of osteoporosis were also analyzed [19]. The possible concurrent medications were defined as medications prescribed at least once during the year 2010. The medical insurance type is divided into national health insurance, which applies to 97% of the population, and medical aid, which applies to patients with income lessthan the minimum cost of living prescribed by law. The regional classification was made by administrative districts consisting of 7 metropolitan cities, including Seoul, and 9 provinces divided by geographic location. For most of the analysis including comorbidity, the unit of analysis was the patient, while for health care institution type and clinician’s specialty, it was the prescription that the patients received.

### Statistical analysis

The differences in age, comorbidity, concurrent medication, osteoporotic fracture, health care institution type, specialty, and prescribed region were compared between bisphosphonate and raloxifene recipients. Medications were quantified in defined daily dose (DDD) in order to make an objective comparison among the study drugs and the different regions. The DDDs of each class of drugs were as follows: 10 mg for alendronate, 5 mg for risedronate, 6 mg for intravenous ibandronate, 5 mg for oral ibandronate, 60 mg for intravenous pamidronate, 4 mg for intravenous zoledronate, and 60 mg for raloxifene HCl [20]. For oral pamidronate, which is currently available in Korea and has no DDD value, 200 was given as the DDD taking into account the recommended daily dose of 200 mg a day. This study presents drug utilization in a standardized unit as DDDs per 1,000 patients per day (DDDs/1,000 patients/day) as recommended by the 2014 WHO Guidelines for ATC classification and DDD assignment. DDDs/1,000 patients/day may provide an estimate of the proportion of population within the patient group treated daily with certain drug. Therefore, for example, 10 DDDs per 1,000 patients per day implies that 1% of the patients on average received a certain drug daily. The DDDs/1,000 patients/day was examined among bisphosphonate by subgroups and different intervals, and the distribution of bisphosphonate and raloxifene prescription was visualized by administrative region. In order to assess the consistency of receiving each drug, switching pattern was analyzed. For comparing bisphosphonate and raloxifene users according to demographic and clinical characteristics, chi-squared tests or Fisher’s exact tests were used for the proportions of categorical variables, and Student’s t-test was used for continuous variables. The trend in the distribution of the proportion by age group was examined using the Cochran–Armitage test for trend. The regional variation of the prescription pattern was geographically visualized by Quantum Geographic Information Systems (QGIS) version 1.6 (OSGeo, Beaverton, OR, USA). Statistical analyses were performed using SAS for Windows version 9.4 (SAS Institute, Cary, NC, USA), and a 2-tailed value of p < 0.05 was considered significant.

## Results

**Fig 1:**
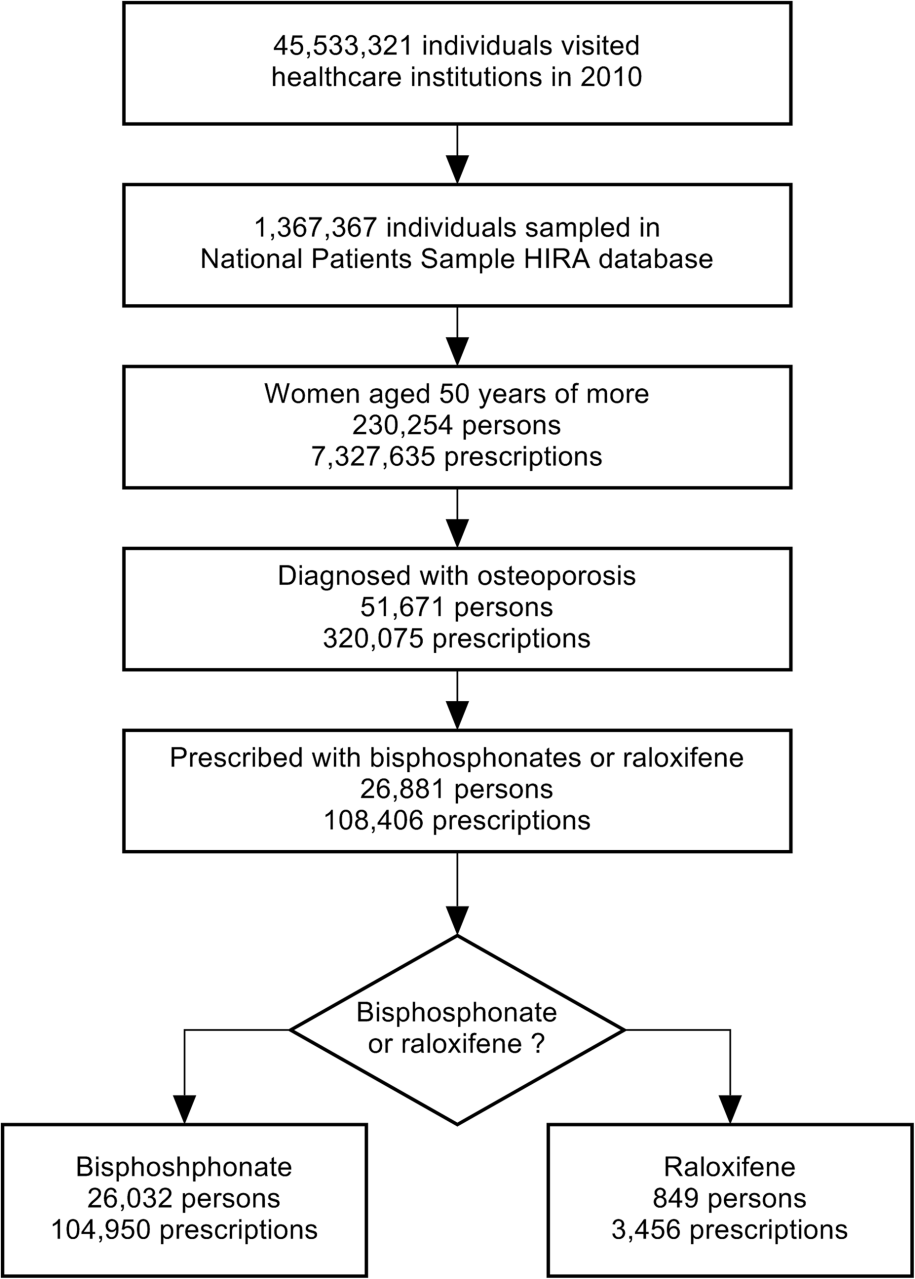
Flowchart of study subject selection. In the 3% sample data of national health insurance claims database, 51,671 were diagnosed with osteoporosis and 26,881 were prescribed with bisphosphonate or raloxifene among them.

Of the 51,671 patients with 320,075 prescriptions who had been diagnosed with osteoporosis by clinicians at least once, 26,881 (52.0%) patients with 108,406 prescriptions (33.9%) had been prescribed with bisphosphonate or raloxifene. Among the 26,881 final study subjects, 26,032 (96.8%) and 849 (3.2%) received bisphosphonate and raloxifene, respectively (Fig 1). The raloxifene users were younger than the bisphosphonate users, and this trend was statistically significant (mean age = 69.3 years vs. 67.6 years, p < 0.0001). The proportion of patients who received bisphosphonate was higher in all three prevalent comorbid conditions; chronic gastritis/GERD (47.3% vs. 42.9%, p = 0.0112), chronic low back pain (39.9% vs. 31.7%, p < 0.0001), and osteoarthritis (29.0% vs. 25.0%, p = 0.0106). However, patients with rheumatic and autoimmune diseases (4.0% vs. 6.1%, p = 0.0024), hot flash (1.8% vs. 6.1%, p <0.0001), coronary artery disease (1.2% vs. 2.8%, p< 0.0001), and lipid metabolism disorders (16.0% vs. 22.1%, p< 0.0001) formed a relatively higher proportion of raloxifene users (Table 1).

**Table 1:** The frequency and proportion of osteoporosis patients by age, comorbidities, fracture by site, concurrent medication, and medical insurance type in Korea in 2010.

	Bisphosphonate		Raloxifene		*p*-value
	N = 26,032		N = 849		
	N	%	N	%	
**Age (Mean ± SD)**	(69.3 ± 8.5)		(67.6 ± 8.7)		<0.0001
50–59	3,750	14.4	168	19.8	<0.0001^a^
60–69	9,235	35.5	336	39.6	
70–79	10,057	38.6	268	31.6	
80–99	2,990	11.5	77	9.1	
**Comorbidities**					
Chronic gastritis/GERD	12,311	47.3	364	42.9	0.0112
Chronic low back pain	10,377	39.9	269	31.7	<0.0001
Osteoarthritis	7,552	29.0	212	25.0	0.0106
Hypertension	6,373	24.5	215	25.3	0.5744
**Vulnerable to osteoporosis**					
Endocrine disorders	3,144	12.1	113	13.4	0.2789
Chronic obstructive lung disease	1,119	4.3	40	4.7	0.5600
Rheumatologic and autoimmune diseases	1,049	4.0	52	6.1	0.0024
Depression	958	3.7	33	3.9	0.7530
Gastrointestinal disorders	348	1.3	12	1.4	0.8484
End-stage renal disease	83	0.3	4	0.5	0.3565^b^
Hypogonadal states	37	0.1	2	0.2	0.3509^b^
**Vulnerable to falling**					
Dementia	486	1.9	18	2.1	0.5925
Parkinson’s disease	327	1.3	15	1.8	0.1914
Epilepsy	136	0.5	15	1.8	<0.0001^b^
**Precautious for raloxifene**					
Stroke	790	3.0	22	2.6	0.4575
Hot flash	469	1.8	52	6.1	<0.0001
Coronary artery disease	323	1.2	24	2.8	<0.0001
Atrial fibrillation or flutter	127	0.5	6	0.7	0.3188 ^b^
Pulmonary embolism/ Deep vein thrombosis/Retinal vein thrombosis	36	0.1	0	0.0	0.6300 ^b^
**Favorable for raloxifene**					
Breast cancer	120	0.5	4	0.5	0.7986^†^
Lipid metabolism disorders	4,178	16.0	188	22.1	<0.0001
**Fracture after prescription by site** ^**c**^					
Vertebra	1,827	7.0	56	6.6	0.7320
Hip	326	1.3	10	1.2	0.8502
Distal radius	279	1.1	8	0.9	0.7201
Humerus	44	0.2	0	0.0	0.4048 ^b^
**Possibly concurrent medication**					
Glucocorticoids	1,911	7.3	66	7.8	0.6344
Anticonvulsants	807	3.1	53	6.2	<0.0001
Proton pump inhibitors	621	2.4	27	3.2	0.1374
Thyroid hormones	492	1.9	29	3.4	0.0015
Selective serotonin reuptake inhibitors	165	0.6	6	0.7	0.7927
Thiazolidinediones	152	0.6	4	0.5	1.0000 ^b^
**Medical insurance type**					
National health insurance	22,724	87.3	764	90	0.0199
Medical aid	3,308	12.7	85	10	

SD, Standard deviation GERD, Gastroesophageal reflux disease

^a^ Cochran–Armitage test for trend analysis

^b^ Fisher’s exact test

^c^ Vertebra (M48.4, M48.5, S22.0, S22.1, S32.0), Hip (S72.0, S72.1), Distal radius (S52.5, S52.6), Humerus (S42.2, S42.3)

Primary care clinics were most frequent in the bisphosphonate group (59.2% vs. 36.7%, p < 0.0001), while tertiary care institutions were most frequent in the raloxifene group (21.6% vs. 44.7%, p < 0.0001). Clinicians in the orthopedics department most frequently prescribed both drugs; however, internal medicine and gynecology specialists prescribed raloxifene more frequently than bisphosphonates for osteoporosis (Table 2).

**Table 2:** The frequency distribution of bisphosphonate and raloxifene prescriptions by health care institution type and specialty in Korea in 2010.

	Bisphosphonate		Raloxifene		*p*-value
	N	%	N	%	
**Health care institutions type** ^**a**^	N = 104,922		N = 3,456		
Primary care clinic	62,158	59.2	1,269	36.7	<0.0001
Secondary care institution	17,003	16.2	599	17.3	0.0772
Tertiary care institution	22,655	21.6	1,543	44.7	<0.0001
Nursing home	547	0.5	32	0.9	0.0013
Public health center	2,559	2.4	13	0.4	<0.0001
**Specialty**	N = 104,950		N = 3,456		
Orthopedics	47,130	44.9	1,287	37.2	<0.0001
Internal medicine	32,058	30.6	1,189	34.4	<0.0001
General physician	6,725	6.4	219	6.3	0.8668
Gynecology	2,012	2.0	258	7.5	<0.0001
Others	17,025	16.2	503	14.6	0.0088

^a^ Of the total 108,406 prescriptions, 28 prescriptions were excluded: 3 prescriptions were missing matching variable for types of health care institutions and 25 prescriptions were issued from oriental medical clinics.

Among bisphosphonates, alendronate was the most frequently prescribed in terms of the number of prescriptions, followed by risedronate, ibandronate, pamidronate, and zoledronate. Nevertheless, the DDDs/1,000 patients/day values revealed that 12.4% of the osteoporosis patients had risedronate daily, 11.6% ibandronate, 11.3% alendronate, 1.6% pamidronate, and 0.4% zoledronate. However, DDDs/1,000 patients/day was the highest for raloxifene, and 15.8% of the osteoporosis patients were prescribed raloxifene daily. Moreover, once a week (79.8%) was the most preferred interval among bisphosphonates (Table 3). Among 26,032 bisphosphonate users, 13,833(53.1%) were prescribed with the drug within 45 days of previous prescription, which was the average day of duration between each prescriptions per patient, and for raloxifene, 447(52.7%) were prescribed within 52days. Among these regular receivers, 94(0.7%) of bisphosphonate users changed to raloxifene and 75(16.8%) vice versa.

**Table 3:** The defined daily dose per 1,000 patients per day (DDDs/1,000 patients/day) of study drugs in Korea in 2010.

	Prescriptions	Patients	DDDs/1,000 patients/day
**Bisphosphonates**	104,950	26,032	
**Raloxifene**	3,456	849	157.6
**Bisphosphonates by subclass**			
Alendronate	54,878	13,141	112.5
Risedronate	40,058	10,005	124.2
Ibandronate	7,833	2,247	115.6
Pamidronate	2,079	578	16.3
Zoledronate	102	61	3.6
**Bisphosphonates by interval**			
Once a week	83,742	20,458	116.0
Once a month	12,235	3,094	170.0
Once a day	5,553	1,210	177.6
Once in 3 months	3,318	1,209	1.3
Once a year	102	61	3.6
**Switch in prescriptions**			
Bisphosphonate→Raloxifene	120	94	-
Raloxifene→ Bisphosphonate	169	75	-

**Fig 2:**
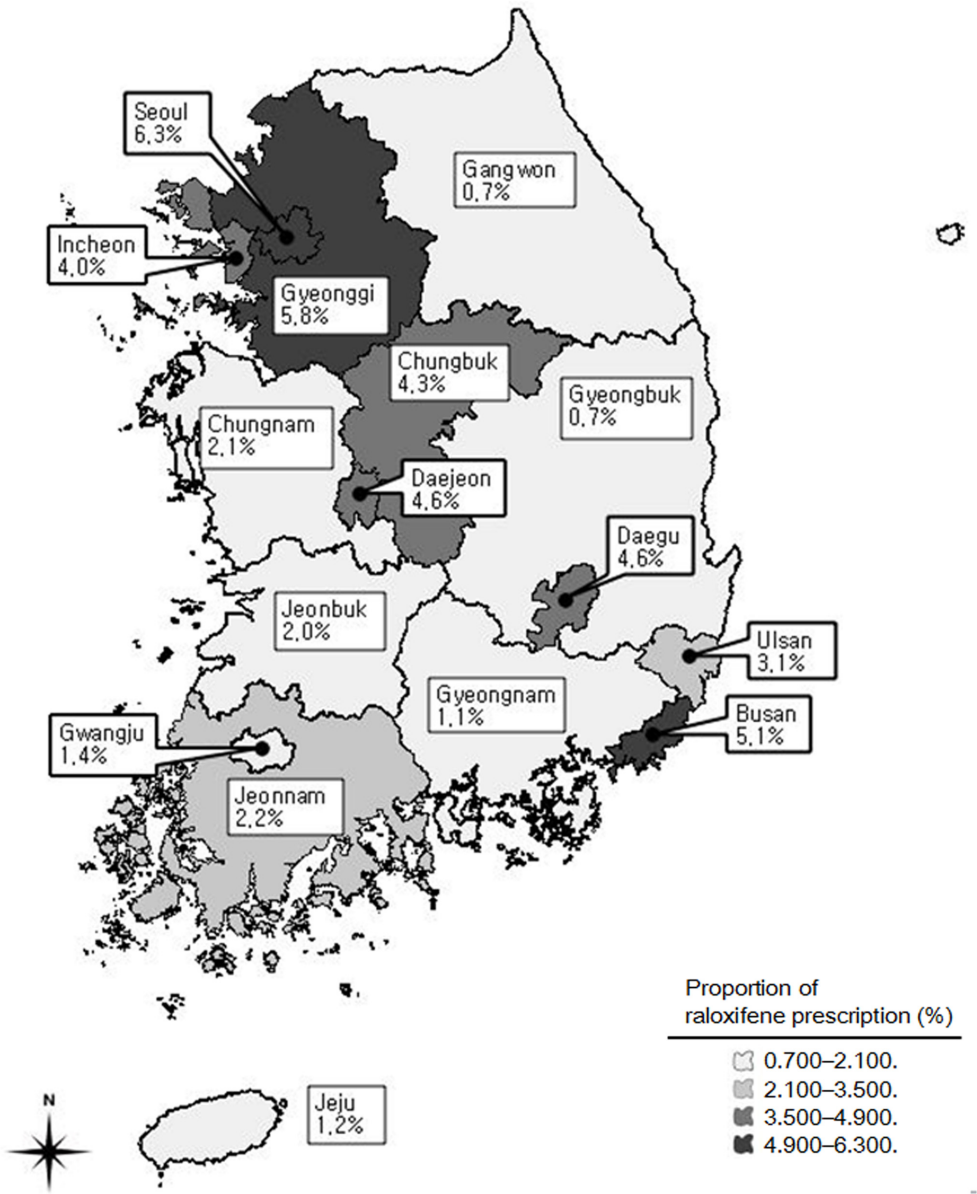
Regional distribution of proportion of raloxifene prescription standardized in DDDs/1,000 patients/day. The Seoul metropolitan area had higher proportion of raloxifene prescriptions among Korean osteoporosis female patients. (The map came from the official site of Statistics Korea, https://sgis.kostat.go.kr/statbd/statbd_03.vw)

However, DDDs/1,000 patients/day examined by different intervals of bisphosphonate was the highest in once a day (17.8%), followed by once a month (17.0%) and once a week (11.6%). The capital area, that is, the Seoul metropolitan area, had the highest DDDs/1,000 patients/day of osteoporosis medication and the highest proportion of raloxifene as compared to the other regions (DDDs/1,000 patients/day = 10.2 in Seoul and 3.5 on average nationwide). Also, metropolitan cities including Busan and Daegu had higher raloxifene prescription than the provinces nearby (Fig 2).

## Discussion

Using a national claims database, we found that 52% of patients diagnosed as osteoporosis were being prescribed with bisphosphonate or raloxifene, two main treatment of osteoporosis prescribed by clinicians. This is similar to 39% taking anti-osteoporosis medication among high risk women from an international cohort, since the risk in this study is from the Fracture Risk Assessment Tool (FRAX) and the diagnosis was made by BMD in the present study [21]. Moreover, bisphosphonate was far more prescribed than raloxifene in Korea, as compared to the relative proportions of the two drugs prescribed in the US. Raloxifene was prescribed less frequently in chronic diseases such as chronic gastritis/GERD, and osteoarthritis, while it was prescribed more frequently in lipid metabolism disorders, hot flash and coronary artery disorders than bisphosphonate. In addition, we observed that raloxifene use was more prevalent in tertiary care institutions and the Seoul metropolitan area.

In this study, 3.2% of osteoporosis patients were prescribed with raloxifene as compared to bisphosphonates. However, raloxifene use was more than six times higher (18.4%) than bisphosphonate use in the US claims data [13]. In a report from Taiwan, which compared alendronate, a type of bisphosphonate, and raloxifene, it was, in fact, ten times higher (32.5%) than our result [9]. The fact that raloxifene is approved for breast cancer risk reduction by the US Food and Drug Administration and not in Korea indicates that bisphosphonate is highly preferred by Korean clinicians for osteoporosis management. Raloxifene users were younger and less frequent in cases of prevalent chronic diseases such as chronic gastritis/GERD, and osteoarthritis, which is in line with the results of a previous study where raloxifene users were younger and had a better overall health status [13]. However, patients with lipid metabolism disorders had higher proportions of raloxifene prescriptions, since they are expected to benefit from raloxifene in their comorbid conditions. Although raloxifene is known to be weaker in decreasing bone turnover markers or fractures, it has been found to be better in improving the lipid profile [5,10,22]. Nevertheless, the proportion of hot flash and coronary artery disease which are well-known adverse drug events of raloxifene were higher among raloxifene users, and this draws an alert for clinicians.

The higher proportion of osteoarthritis in the bisphosphonate group correlated well with the higher proportion of bisphosphonate from orthopedics clinicians in primary care institutions who also frequently treat osteoporosis and are more familiar with bisphosphonates than raloxifene. Likewise, gynecologists who are more aware of hormone replacement therapies and female organ specific cancers were more likely to prescribe raloxifene than bisphosphonates, as in Foster’s research [14]. However, the raloxifene group had a higher prevalence of rheumatologic and autoimmune diseases including rheumatoid arthritis, ankylosing spondylitis, and lupus, which is in contrast to the findings of a previous study [13]. This can be explained by the higher proportion of tertiary health care institutions (57.8%, not presented in the table) among these patients because raloxifene was more commonly prescribed by clinicians in the tertiary referral hospitals. The interest in trying and accepting a relatively new and challenging drug might be higher in doctors of tertiary health care institutions than those at primary clinics. Even though previous studies do not show the difference according to the level of health institutions, our study showed these patterns comprehensively.

Bisphosphonate, but not raloxifene, is recommended for prevention of corticosteroid-induced osteoporosis, a condition taken into account when corticosteroids are prescribed for more than three months. However, the use of corticosteroid did not differ between the two groups in this study. Among the corticosteroid prescribers, patients with diagnosis of secondary osteoporosis (ICD-10 code: M82) were nine and among them only one patient was prescribed with raloxifene who also had gastritis. All of the corticosteroid users were prescribed within less than 50 days and 72.5% were prescribed less than 3 times. Thus, even though corticosteroid is a well-known risk for osteoporosis, majority of them were not recognized as corticosteroid-induced osteoporosis but primary osteoporosis. However, among patients who had osteoporotic fracture during 2010, there was no one who had been prescribed with bisphosphonate or raloxifene before their fracture occurred. Among 2,550 fracture events, 2,496 patients had at least one fracture and 2,000 (81.0%) started to receive either bisphosphonate or raloxifene at the same time with osteoporotic fracture incidence. It is reported from a national survey that among Korean osteoporosis patients over 50 years old, 23.5% receive pharmacological treatment [23]. Low treatment rate thus explains the lack of patients who were receiving bisphosphonate or raloxifene before fracture actually happened. This raises an attention in the low treatment rate of osteoporosis and its negative consequences once again.

In terms of DDDs/1,000 patients/day, raloxifene was the highest in total, and risedronate was the highest among bisphosphonates. Surprisingly, although only 3% of the patients were prescribed raloxifene, it had the highest DDDs/1,000 patients/day among the study drugs. This implies a good adherence to raloxifene, same as in a previous study which reported that raloxifene had better adherence and more patient satisfaction than bisphosphonate [24]. In number of prescriptions, alendronate and once-a-week basis were the most preferred treatment. However, in terms of DDDs/1,000 patients/day, risedronate and once-a-day basis had the highest ranking. This must have resulted from the dominantly high number of recipients in alendronate or once-a-week basis because DDDs/1,000 patients/day is calculated by dividing the patient number of each drug. To sum up, even though alendronate was the most favored prescription, risedronate was the most adherent one, and while once-a-week basis was more commonly prescribed, once-a-day basis was more compliant. Furthermore, alendronate and risedronate are known to have similar absolute fracture rates [25]. Among regular receivers of bisphosphonate or raloxifene, while less than 1% of bisphosphonate users were switched to raloxifene, 17% changed from raloxifene to bisphosphonate. Among these switchers of raloxifene, 33 patients were due to visiting another specialty for arthritis or intervertebral disc disorders which were mainly orthopedics. Also, nine patients changed to bisphosphonate due to osteoporotic fracture, and two patients due to diabetes which is a risk factor for stroke. Therefore, switching from raloxifene to bisphosphonate seemed to take place mainly owing to clinicians’ preference determined by their specialties. This could be a problem in the consistency of treatment since patients tend to change their hospitals often without a family doctor system in Korea.

While there was a large regional variation, DDDs/1,000 patients/day was higher near the capital area. In Korea, the Seoul metropolitan area including Seoul and Gyeonggi is considered to have the highest level of economic status in the country. The management of osteoporosis by clinicians must have been more active by clinicians in the capital area, suggesting more attention in the rural area, where the prevalence of osteoporosis is known to be higher [23]. It is said that a physician-specific tendency was the strongest determinant in osteoporosis patient care variation [26]. Furthermore, the physician factor is known to be affected by academic activities conducted by pharmaceutical companies or the physician’s number of years of practice [27]. However, in this database, 42.3% of the health care institutions were tertiary referral hospitals in the Seoul metropolitan area, which tend to exist in economically developed regions. This has resulted in a high proportion of raloxifene prescriptions in these regions, implying that patient care can be somewhat different among regions due to the different distribution of health care institutions nationwide.

The strength of this study comes from the nationally representative database. HIRA-NPS is sampled from the nationwide health insurance claims database, which consists of 97% of the whole 50 million Korean population [28]. The representativeness and the effectiveness of the healthcare utilization database in studying the real world are well known [29], and the validity and the generalizability of the HIRA-NPS database have been assessed in advance [17]. Therefore, this result can be generalizable to the entire population. Second, since this database is formed in the process of reimbursement, a misclassification of the prescribed drugs is less likely to happen, reflecting the actual clinical practice taking place in the clinics [29]. However, this study has some limitations. First, it lacks laboratory examination data such as BMD, bone turnover markers, or body mass index, and a consideration of lifestyle factors such as smoking, alcohol consumption, or diet. However, even though BMD is used in the diagnosis of osteoporosis, it alone is considered imperfect for predicting fracture vulnerability [30]. Moreover, in the case of vertebral fractures, factors associated with behavioral patterns such as obesity, alcohol intake, or smoking have been found to have little or no effect among Koreans [31].

In conclusion, with a high preference for bisphosphonate in osteoporosis management in Korea, bisphosphonate and raloxifene users among Korean women had different patterns of prescription in different comorbid conditions, concurrent medications, health care institution types, clinicians’ specialties, and regions. The potential adverse drug events of bisphosphonate and raloxifene such as gastrointestinal disorders, coronary artery disease, or hot flash should be examined prior to giving prescriptions. However, we cannot draw a conclusion on the appropriateness of this clinical practice since the current guidelines suggest these two medications not strictly for specific situations but as generic medications for osteoporosis. Thus, it is required to conduct a long- term comparative effectiveness study in order to provide a detailed guide for the appropriate management of osteoporosis.
